# Neutrophil to lymphocyte ratio as an assessment tool to differentiate between uterine sarcoma and myoma: a systematic review and meta-analysis

**DOI:** 10.1186/s12885-023-11775-5

**Published:** 2024-01-02

**Authors:** Fatemeh Tabatabaei, Saghar Babadi, Shima Nourigheimasi, Arshin Ghaedi, Monireh Khanzadeh, Aida Bazrgar, Morad Kohandel Gargari, Shokoufeh Khanzadeh

**Affiliations:** 1 Department of Obstetrics and Gynaecology, School of Medicine, Tabriz University of Medical Sciences, Tabriz, Iran; 2 Department of Gynaecologic Laparoscopic Surgeries, Al-Zahra Hospital, Tabriz University of Medical Sciences, Tabriz, Iran; 3 Ahvaz Jundishapur University of Medical Science, Ahvaz, Iran; 4 School of Medicine, Arak University of Medical Sciences, Arak, Iran; 5 Student Research Committee, School of Medicine, Shiraz University of Medical Sciences, Shiraz, Iran; 6 Trauma Research Center, Shahid Rajaee (Emtiaz) Trauma Hospital, Shiraz University of Medical Sciences, Shiraz, Iran; 7 Geriatric & Gerontology Department, Medical School, Tehran University of medical and health sciences, Tehran, Iran; 8 Tabriz University of Medical Sciences, Tabriz, Iran

**Keywords:** Neutrophil to lymphocyte ratio, NLR, Uterine sarcoma, Uterine leiomyoma, Meta-analysis

## Abstract

**Background:**

This systematic review and meta-analysis aimed to determine the potential value of neutrophil to lymphocyte ratio (NLR) as an assessment tool in the clinical distinction between uterine sarcoma and uterine leiomyoma.

**Methods:**

We comprehensively searched Web of Science, Scopus, and PubMed for relevant papers published before March 19, 2023. The standardized mean difference (SMD) was provided, along with a 95% confidence interval (CI). The random-effects model was employed to derive pooled effects due to the high levels of heterogeneity. The Newcastle-Ottawa scale was used for the quality assessment. Our study was registered in PROSPERO (CRD42023478331).

**Results:**

Overall, seven articles were included in the analysis. A random-effect model revealed that patients with uterine sarcoma had higher NLR levels compared to those with uterine myoma (SMD = 0.60, 95% CI = 0.22–0.98; *p* = 0.002). In the subgroup analysis according to sample size, we found that patients with uterine sarcoma had elevated levels of NLR compared to those with uterine myoma in either large studies (SMD = 0.58, 95% CI = 0.04–1.13; *P* < 0.001) or small studies (SMD = 0.64, 95% CI = 0.33–0.96; *P* = 0.32). In the sensitivity analysis, we found that the final result was not significantly changed when single studies were removed, suggesting that the finding of this meta-analysis was stable. The pooled sensitivity of NLR was 0.68 (95% CI = 0.61–0.73), and the pooled specificity was 0.64 (95% CI = 0.59–0.69).

**Conclusion:**

NLR might be utilized as an assessment tool in clinics to help clinicians differentiate between patients with uterine sarcoma and those with myoma.

**Supplementary Information:**

The online version contains supplementary material available at 10.1186/s12885-023-11775-5.

## Background

Every gynecologist faces uterine tumors as a serious issue in their clinical work. The most frequent benign tumor of the female reproductive system is uterine myoma. Myomas, which appear as single or multiple lesions, are among the most frequent disorders in gynecological patients due to their high frequency in the population and their harmful effects on health [1, 2]. On the other hand, uterine sarcoma, which accounts for 3–7% of all malignant uterine tumors, has an incidence of 0.7 per 100,000 [3]. The most aggressive form of uterine sarcoma is leiomyosarcoma (LMS) which has a poor prognosis even in stage I (5-year survival rate = 50%) [4]. Accurate diagnosis and management of uterine sarcoma face significant challenges as it can be misdiagnosed as a benign tumor [2]. For instance, differentiating uterine sarcoma from uterine leiomyoma is one of the main issues. Although magnetic resonance imaging (MRI) is frequently utilized to make the correct diagnosis, it is still challenging to distinguish uterine sarcoma from uterine leiomyoma [5]. So, there is a need for an inexpensive biomarker alongside MRI for the correct differentiation between these two tumors, and neutrophil to lymphocyte ratio (NLR) is one of them. NLR, a serum indicator of systemic inflammation that is simple to test, has been studied as an effective prognostic or diagnostic biomarker in several malignancies and gynecological diseases, such as colorectal cancer, non-small cell lung cancer (NSCLC), endometriosis, and pelvic inflammatory disease [6–9]. Furthermore, the effectiveness of NLR in distinguishing uterine sarcoma from leiomyoma has been studied in previous original studies, but the overall results were controversial [10–16].

So, a systematic review and meta-analysis is needed to pool the results from previous studies and clarify the effectiveness of NLR in distinguishing uterine sarcoma from leiomyoma. This way, NLR may be used as a simple assessment tool to guide clinicians to intervene early and enhance patient outcomes. This systematic review and meta-analysis aims to review the evidence on the role of NLR in differentiating uterine sarcoma from uterine leiomyoma.

## Methods

### Eligibility criteria

The Preferred Reporting Items for Systematic Reviews and Meta-Analyses (PRISMA) was used to conduct this systematic review. This study was registered in the Prospective Register of Systematic Reviews (PROSPERO) with ID of CRD42023478331. Based on PICO, we included human studies that met the following eligibility criteria:

- Population: Patients with uterine sarcoma
- Intervention/Exposure. NLR level
- Control. Patients with uterine myoma
- Outcomes. NLR as an assessment tool
- Study Design. Cross-sectional or case-control studies were included in our review. However, our search strategy was not limited to any specific study design.

### Screening of studies

Literature searches of the PubMed, Scopus, and Web of Science (WOS) databases were performed to find publications reporting NLR measures for patients with uterine sarcoma and myoma from inception to March 19, 2023. No restrictions were applied regarding the data or language. The same search approach was employed: “((neutrophil to lymphocyte ratio) OR NLR) AND uterine AND myoma AND sarcoma.” According to the inclusion criteria, articles with possibly relevant abstracts and titles were included. These publications were simultaneously assessed for study types, correct interventions, and outcomes to establish eligibility for full-text review. Data extraction was performed on included full-text articles. When disagreements over study selection developed, a third reviewer served as a mediator.

### Qualitative analysis

The Newcastle-Ottawa scale (NOS) was employed to evaluate the risk of bias. The NOS criteria allowed for a maximum of two stars in comparability, four stars in the selection, and three stars in the outcome, with an overall score ranging between 0 to 9.

### Data charting process and data items

Three reviewers gathered data independently. Author, study type, publication year, country of research, number of patients in sarcoma and myoma groups, and mean and standard deviation (SD) on NLR level in each group were among the data elements obtained.

### Statistical analysis

Stata 14 (STATA Corp., College Station, TX, USA) was used to analyse the data. Effect sizes were expressed using the standardized mean difference (SMD) by meta-analysis. A *P* value < 0.05 was regarded to be significant. The standardized mean difference was used in the meta-analysis to represent effect sizes (SMD). A P value < 0.05 was deemed significant. Due to the low number of articles in the meta-analysis, heterogeneity between studies was determined using I^2^, derived from Cochran’s Q. A random-effects model was chosen if I^2^ was more than 50%.

Moreover, we utilized sensitivity analysis to determine the impact of a single study on the total mean difference. According to the sample size, subgroup analysis was done.

The diagnostic odds ratio (DOR), positive likelihood ratio, pooled specificity, specificity, and negative likelihood ratio were calculated using the “metandi” command. In addition, a summary receiver operating characteristic (SROC) curve was created.

## Results

### Search results and included studies

**Fig. 1:**
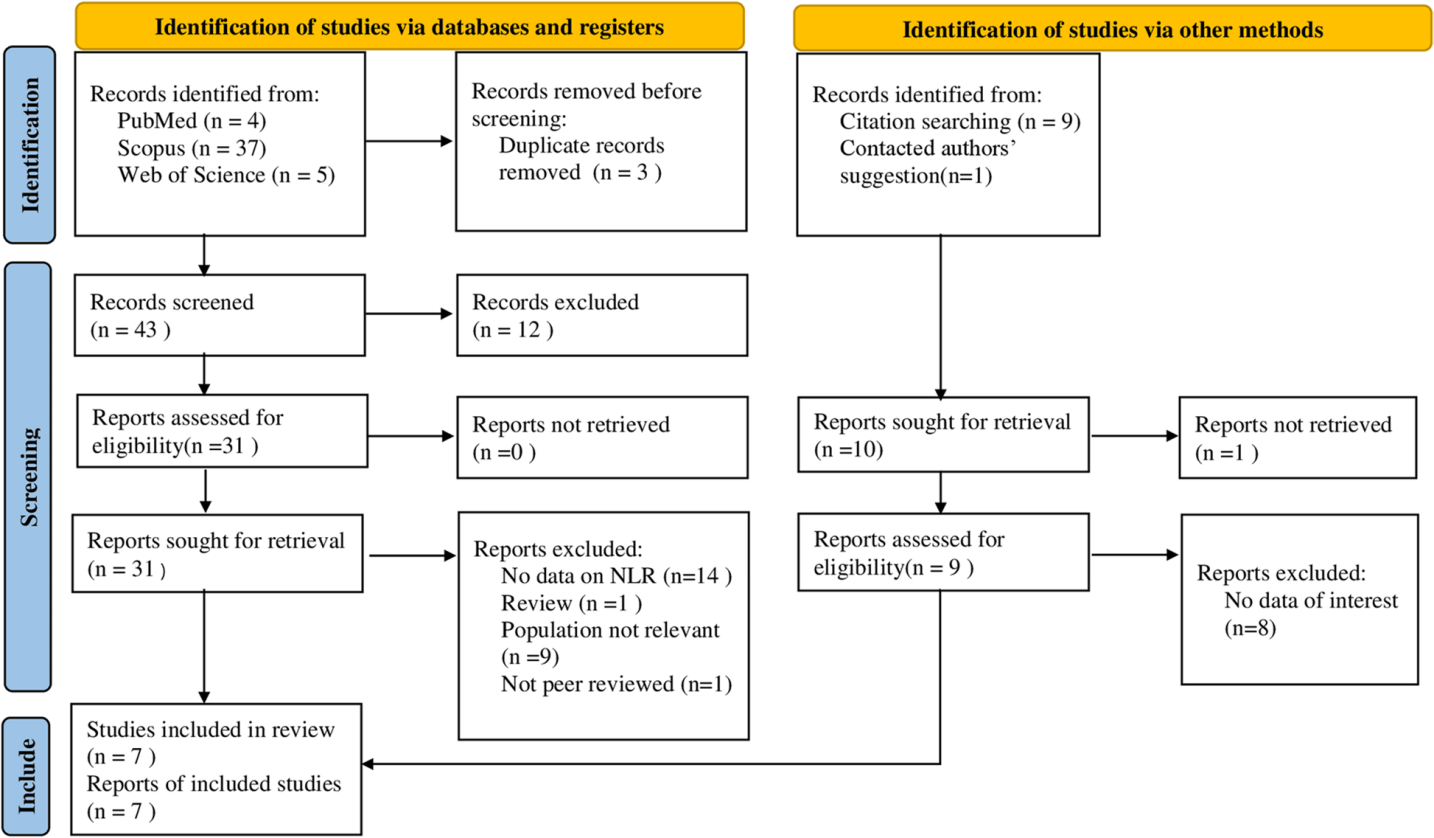
PRISMA 2020 Flow diagram for new systematic reviews, which includes searches of databases, registers, and other sources

Figure 1 represents the study selection process. The first literature search yielded 56 papers for consideration. Our systematic review and meta-analysis included seven papers after numerous phases of screening.

### Characteristics of the population and quality assessment

Seven articles were included in the analysis [10–16], including 1213 patients with uterine myoma and 319 patients with uterine sarcoma. Six of them compared patients with uterine myoma and sarcoma [10–12, 14–16], and five reported NLR’s sensitivity and specificity in differentiating between patients with uterine myoma and sarcoma [10–13, 15]. Table 1 shows the overall characteristics and quality scores of the included articles.

**Table 1:** General characteristics of included studies

Author	Year	Country	Design	Sarcoma		Myoma		Cut off point	Sensitivity	Specificity	NOS Score
				N	NLR	N	NLR				
Kim	2010	South Korea	Retrospective	55	4.59 ± 5.73	165	2.05 ± 1.87	2.12	74.5	70.3	7
Yeon	2012	South Korea	Retrospective	34	3.70 ± 4.87	34	1.90 ± 1.13	–	–	–	7
Cho	2015	South Korea	Retrospective	31	3.90 ± 5.06	93	1.90 ± 1.16	2.1	43.2	82.8	6
Zhang	2020	China	Retrospective	45	3.30 ± 2.50	180	2.30 ± 1.10	–	–	–	7
Jeong,K.	2021	South Korea	Retrospective	40	3.90 ± 3.80	326	2.00 ± 1.20	2.6	60	83.4	8
Suh	2021	South Korea	Retrospective	79	–	257	–	2.15	63.5	61.7	8
Aksakal	2022	Turkey	Retrospective	35	2.97 ± 1.06	158	3.42 ± 1.86	2.04	59.4	59.5	7

*N* Number, *NLR* Neutrophil to lymphocyte ratio

### Differences in NLR level between patients with endometriosis and healthy controls

**Fig. 2:**
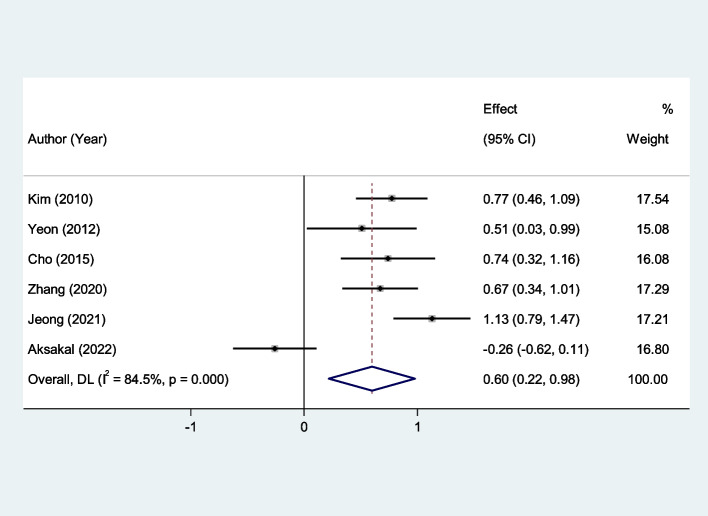
Meta-analysis of differences in NLR level between patients with uterine sarcoma and those with uterine myoma

Patients with uterine sarcoma had elevated levels of NLR compared to those with uterine myoma (SMD = 0.60, 95% CI = 0.22–0.98; *p* = 0.002; Fig. 2).

**Fig. 3:**
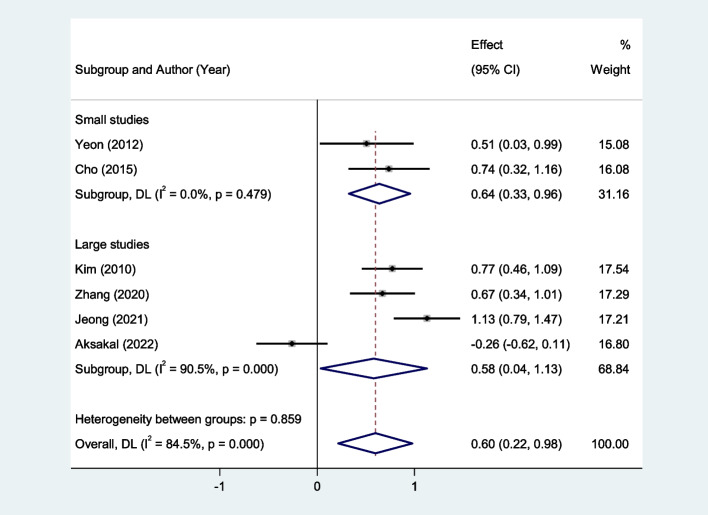
In the subgroup analysis of differences in NLR level between patients with uterine sarcoma and those with uterine myoma, according to sample size

In the subgroup analysis according to sample size, we found that patients with uterine sarcoma had elevated levels of NLR compared to those with uterine myoma in either large studies (SMD = 0.58, 95% CI = 0.04–1.13; *P* < 0.001) or small studies (SMD = 0.64, 95% CI = 0.33–0.96; *P* = 0.032, Fig. 3).

### Sensitivity analysis

**Fig. 4:**
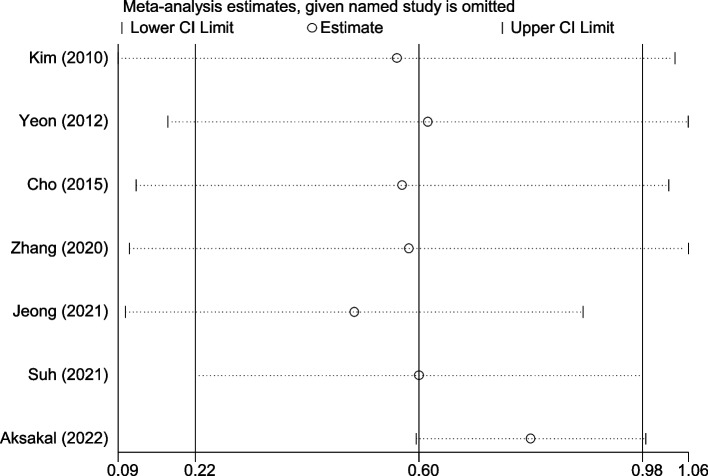
Sensitivity analysis of differences in NLR level between patients with uterine sarcoma and those with uterine myoma

In the sensitivity analysis, we found that the final result was not significantly changed when single studies were removed, suggesting that the finding of this meta-analysis was stable (Fig. 4, Table S1).

### NLR’ value in differentiating between tumors

**Fig. 5:**
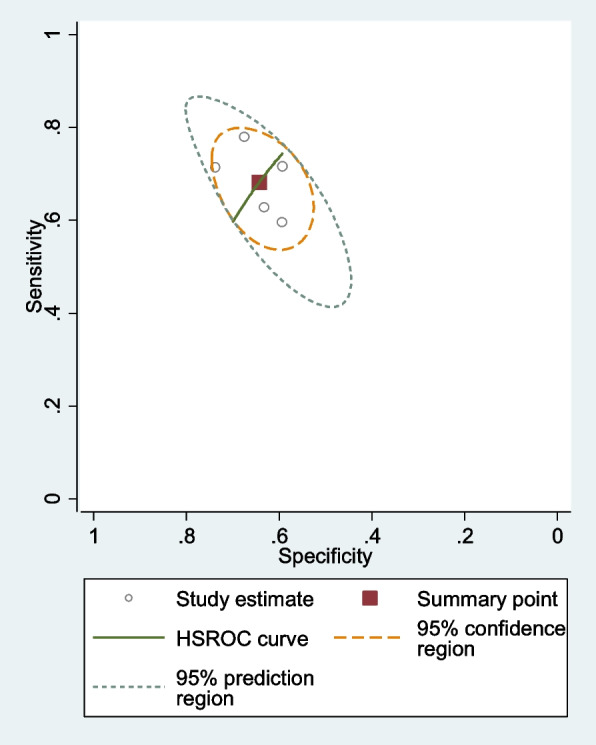
SROC curve of included studies assessing diagnostic value of NLR for uterine tumors

The pooled sensitivity was 0.68 (95% CI = 0.61–0.73), and the pooled specificity was 0.64 (95% CI = 0.59–0.69). The pooled positive likelihood ratio, negative likelihood ratio, and diagnostic odds ratio (DOR) of NLR were 1.91(95%CI = 1.57–2.31), 0.49 (95%CI = 0.39–0.62), and 2.02 (95%CI = 1.60–2.54), respectively (Fig. 5). By summing the specificity and sensitivity of NLR in each study, we found that the best cut-off point for NLR was 2.12 according to Kim et al. [12].

### Publication bias

**Fig. 6:**
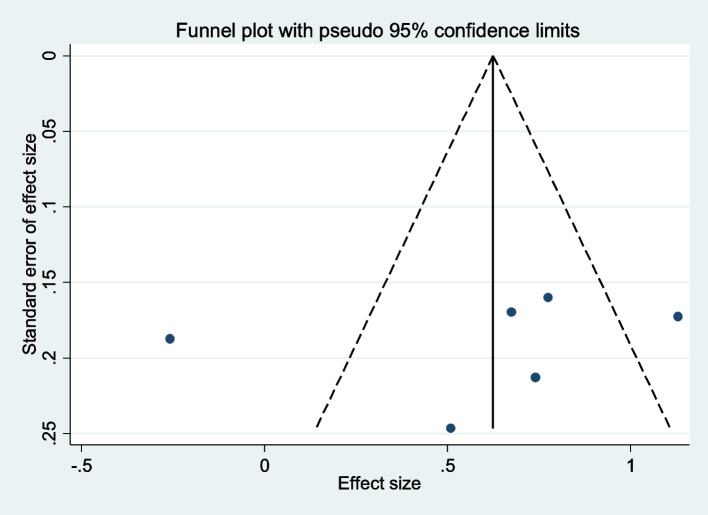
Funnel plot assessing publication bias

As seen in Fig. 6, there was no publication bias among included studies (Egger test *P* = 0.63).

## Discussion

The current systematic review and meta-analysis were carried out to examine the potential of NLR as an assessment tool in uterine sarcoma. The main findings of our research were as follows: Patients with uterine sarcoma had elevated levels of NLR compared to those with uterine myoma. In the subgroup analysis, according to sample size, patients with uterine sarcoma had elevated levels of NLR compared to those with uterine myoma in either large or small studies. The pooled sensitivity of NLR was 0.68 (95% CI = 0.61–0.73), and the pooled specificity was 0.64 (95% CI = 0.59–0.69).

Most clinicians find it difficult to differentiate between myoma and sarcoma before surgery. Since the preoperative differential diagnosis of sarcoma and myoma is challenging, patients are usually diagnosed with a final pathological biopsy after surgery. If a suspected leiomyoma is later revealed as a uterine sarcoma, the morcellation performed in surgery leads to a poor prognosis. As a result, motorized morcellation in robotic or laparoscopic myomectomy is debatable [17, 18]. If myomectomy is scheduled for minimally invasive surgery, distinguishing between uterine sarcoma and myoma prior to surgery is critical. As a result, numerous imaging techniques, like MRI, pelvic ultrasonography, and PET-CT, have been used to discriminate between myomas and sarcomas before surgery. According to a research by Li et al. [19], MRI had a 100% sensitivity rate and a 90% specificity rate for differentiating between sarcomas and degenerated myomas. Thus far, the most helpful preoperative imaging test is MRI. However, it may not be cost-effective to undergo an MRI on every suspected myoma patient. Hence, pelvic ultrasonography should be preferred due to its ease of use, and MRI should be done when sarcomas are suspected due to ultrasonographic results. Before undertaking pricey tests like MRI and PET-CT, there is a need for other techniques that may be employed to help with ultrasound exams. Also, it could be beneficial if other techniques might aid in differential diagnosis before undergoing expensive imaging when sarcoma is suspected following ultrasonography. The CBC with differential counts, which is often conducted preoperatively in nearly all patients, is the simplest, fastest, and easiest technique to get findings [15].

It is now widely acknowledged that cancer and inflammation are closely related, and growing studies suggest that chronic inflammation contributes significantly to therapeutic response, tumor progression, carcinogenesis, and clinical outcome [20, 21]. Hence, indicators of systemic inflammation may provide insightful data concerning the occurrence of malignancy.

LMS is characterized by hemorrhage and tumor necrosis, and the latter is linked to local inflammation, [22] suggesting that serum markers may reflect these circumstances. Additionally, some hematological alterations, like an increase in neutrophil count and a decrease in lymphocyte count, are seen in cancer patients. It has been proven that neutrophils play a part in the connection between cancer and inflammation, as well as the development of a tumor microenvironment that promotes metastasis, cancer progression, and angiogenesis [23]. On the other hand, lymphocytes are engaged in the cell-mediated response to tumor infiltration, and reduced lymphocyte count may lead to an insufficient immune response, which is linked with adverse results [24].

High neutrophil levels in tissues secrete numerous inflammatory mediators like vascular endothelial growth factor (VEGF), tumor necrosis factor-a (TNF-a), interleukin-2 (IL-2), interleukin-10 (IL-10), and interleukin-6 (IL-6) which creates a suitable environment for cancer progression [25–27]. Moreover, producing several cytokines and chemokines by neutrophil infiltration may inhibit the immunological function of lymphocytes and natural killer cells [25, 28]. Lymphocytes are important components of the host immunological response. By triggering cytokine production and cytotoxic cell death, they may reduce the capacity of cancer cells to proliferate and metastasize [29]. Tumor-infiltrating lymphocytes (TILs) have a role in several phases of tumor progression [30, 31]. An increasing amount of data suggests that tumor-infiltrating CD4+ and CD8+ T cells may be a prognostic biomarker in various cancers [32–34]. As a result, NLR may reflect a balance between tumor development and antitumor immune activity [35].

According to the latest studies, NLR may be a helpful diagnostic and prognostic marker for several malignancies [24, 36–40]. A high NLR shows an increased immunosuppressive state and is related to a worse prognosis for gastric, breast, esophageal, urologic, lung, and colorectal malignancies [36, 38]. Regarding gynecologic cancers, high NLR is linked to poor clinical results in endometrial, ovarian, cervical cancer, and uterine sarcoma [24, 36, 37, 39]. A higher NLR was strongly related to poor clinical outcomes and adverse clinicopathological variables in Wu et al.’s meta-analysis of cervical cancer [40].

Similarly, Jeong et al. observed that patients with uterine sarcoma had poor clinical outcomes when their preoperative NLR was higher (≥2.60) [24]. Nevertheless, few studies have examined systemic inflammation markers’ diagnostic significance in various malignancies. Kim et al. reported the usefulness of NLR for the preoperative diagnosis of uterine sarcomas and proposed that NLR (≥2.12) would be a more practical and cost-effective measure of preoperative differentiation than serum CA-125 [12].

Cho et al. found that an NLR of > 2.1 independently and significantly indicated the existence of uterine sarcoma [11], while Zhang et al. showed that an NLR of ≥2.8 independently indicated LMS [14]. Similarly, Suh et al. discovered that an NLR of ≥2.157 may effectively distinguish LMS from LM [13]. There may be markers similar to NLR which have been used as prognostic indicators in gynecological diseases. For instance, Peker et al. showed that red blood cell distribution width coefficient of variation (RDW-CV) can predict clomiphene citrate resistance (CC-R) in anovulatory, infertile women suffering from polycystic ovarian syndrome (PCOS) [41].

### Limitations and strengths

Our research has a few limitations that should be explained. The primary drawback of this study is the limited number of papers included in this review. As a result, the strength of our results may be jeopardized, and further research will be required to strengthen our findings. The research included in our analysis also showed substantial heterogeneity. High heterogeneity may still be a concern even if the random effect model was employed to account for it. Also it is important to note that all included studies were not randomized prospective studies and they have their limited flaws. Finally, it is noteworthy to mention that NLR is more an assessment tool than a diagnostic test which could potentially complement other preoperative assessment tools of uterine myomas to predict patients who might have uterine sarcomas. It is important to stress that despite all our efforts using imaging and other methods, diagnosis of uterine sarcomas cannot be ruled out with 100% certainty. Nonetheless, our systematic search, which was supplemented by a thorough review of the references in the retrieved papers, is a critical strength of our study. To the best of our knowledge, this was the first meta-analysis that investigated the role of NLR in distinguishing between uterine sarcoma and myoma.

## Conclusion

In this systematic review and meta-analysis, we observed that patients with uterine sarcoma had higher levels of NLR than those with uterine myoma. As a result, our data imply that the NLR has an underlying effectiveness in predicting uterine sarcoma. This study recommends that patients with a NLR value above 2.12 should be referred to the gynecological oncological surgeons in the tertiary center. While discrepancies in NLR prediction between uterine sarcoma and myoma may be attributed to varying degrees of immunosuppression or cytokine expression changes by different tumor microenvironments, the NLR seems predictive for uterine sarcoma. The findings of this research may verify NLR as an assessment tool for sarcoma while also clarifying pathophysiology to aid in treatment development. Further study is required to determine the sensitivity and specificity of NLR as prognostic biomarkers in sarcomas.

### Supplementary Information

**Additional file 1: Table S1. **The results of sensitivity analysis.

## Data Availability

All data generated or analysed during this study are included in this published article.

## Abbreviations

NLR: Neutrophil to lymphocyte ratio
SMD: Standardized mean difference
CI: Confidence interval
LMS: Leiomyosarcoma
MRI: Magnetic resonance imaging
NSCLC: Non-small cell lung cancer
PRISMA: Preferred Reporting Items for Systematic Reviews and Meta-Analyses
WOS: Web of Science
NOS: Newcastle-Ottawa scale
SD: Standard deviation
DOR: Diagnostic odds ratio
SROC: Summary receiver operating characteristic
VEGF: Vascular endothelial growth factor
TNF-a: Tumor necrosis factor-a
IL-2: Interleukin-2
IL-10: Interleukin-10
IL-6: Interleukin-6
TILs: Tumor-infiltrating lymphocytes
