# Supplementary material for: Neutrophil to lymphocyte ratio as an assessment tool to differentiate between uterine sarcoma and myoma: a systematic review and meta-analysis
Source: BMC Cancer. 2024 Jan 2;24:12. doi: 10.1186/s12885-023-11775-5 (PMC10763287; doi:10.1186/s12885-023-11775-5)
Supplement: Supplementary file 1 — Additional file 1: Table S1. The results of sensitivity analysis. [file 12885_2023_11775_MOESM1_ESM.docx]

**Differences in neutrophil to lymphocyte ratio between patients with uterine sarcoma and those with myoma: A meta-analysis**

**Table S1.** The results of sensitivity analysis

------------------------------------------------------------------------------

Study omitted | Estimate [95% Conf. Interval]

-------------------+----------------------------------------------------------

Kim (2010) | .56089562 .08587576 1.0359155

Yeon (2012) | .61436617 .17090994 1.0578225

Cho (2015) | .57086754 .11711883 1.0246162

Zhang (2020) | .58221322 .10565707 1.0587693

Jeong (2021) | .48890969 .09871639 .879103

Suh (2021) | .59885764 .21775666 .97995865

Aksakal (2022) | .78982949 .59401977 .98563927

-------------------+----------------------------------------------------------

Combined | .59885766 .21775666 .97995866

------------------------------------------------------------------------------
